# HPLC analysis, genotoxic and antioxidant potential of *Achillea millefolium* L. and *Chaerophyllum villosum* Wall ex. Dc

**DOI:** 10.1186/s12906-024-04344-1

**Published:** 2024-02-16

**Authors:** Muhammad Adil, Ghulam Dastagir, Atifa Quddoos, Muhammad Naseer, Faten Zubair Filimban

**Affiliations:** 1 Pharmacognosy Laboratory, Department of Botany, University of Peshawar, Peshawar, Pakistan; 2 Centre for Plant Sciences and Biodiversity, University of Swat, Charbagh, Pakistan; 3 Department of Chemical and Life Sciences, Qurtuba University of Science and Information Technology, Peshawar, Pakistan; 4 King Abdulaziz University, Faculty of Science Department of Biological Sciences, Division of Botany, Jeddah, Saudi Arabia

**Keywords:** Phytochemical screening, HPLC, Genotoxic activity, Antioxidant, Methanolic extract, Chloroformic extract, Prevention, *Achillea millefolium*, *Chaerophyllum villosum*

## Abstract

**Background:**

Methanolic and chloroformic extract of *Achillea millefolium* and *Chaerophyllum villosum* were evaluated for HPLC analysis, genotoxic and antioxidant potential.

**Materials and methods:**

Genotoxic activity was carried out on human blood lymphocytes via comet assay and antioxidant activity was studied through DPPH method.

**Results:**

The genotoxic potential of *A. millefolium* and *C*. *villosum*’s methanolic and chloroformic extract was analysed using comet assay technique. Comet shaped human lymphocytes cells were observed when treated with different concentrations (50 mg/mL, 75 mg/mL, 100 mg/mL) of methanolic and chloroformic extract of both plants. Reading was taken on the basis of damaged DNA head and tail length. Greater the length of tail as compared to head, greater will be the damage and vice versa. Total comet score was obtained from *A. millefolium* subjected to different concentrations. After a time interval of 24 h both the extract showed dose dependant genoprotection with maximum genoprotectivity at 98.7 ± 12.7 and 116 ± 5.3 at 50 mg/100 mL for methanolic and chloroformic extract respectively. Similarly Total Comet score was obtained from *C. villosum* subjected to different concentrations of methanolic and chloroformic extract. After 24 h exhibited dose dependent genoprotection with maximum protectivity at 85.7 ± 22.0 and 101.7 ± 8.6 at 50 mg/100 mL for methanolic and chloroformic extract were determined. The antioxidant activity revealed that methanolic extract of *A. millefolium* showed highest antioxidant activity (84.21%) at 300 mg/ml after 90 min while the chloroformic extract of *C. villosum* exhibited highest (68.46%) antioxidant activity (59.69%) at 300 µg/ml after 90 min but less than the standard drug ascorbic acid (88.72%). Quantitative phytochemical screening revealed high percentage of alkaloids (27.4%), Phenols (34.5%), Flavonoids (32.4%) as compared to Tannins (12%) in methanolic extract of *A.millefolium.* While high percentage of alkaloids (31.4), Phenols (19.3%), Flavonoids (35.5%) as compared to Tannins (16.6%) in chloroformic extract of *C. villosum.*

**Conclusion:**

The present results showed that A. *millefolium* and *C. villosum* possess a number of important compounds and revealed genoprotective property which may be used to treat several genetic disorders such as alzeimer’s disease in future (Grodzicki W, Dziendzikowska K, Antioxidants 9(3):229, 2020).

## Background

Numerous physiological and biochemical processes in the human body and environmental factors may produce oxygen-centered free radicals and other reactive oxygen species as by products. Overproduction of free radicals can cause oxidative damage to biomolecules in the body, such as lipids, proteins and DNA [1]. In human body these free radicals can be scavenged by several enzymatic and non-enzymatic antioxidant defence mechanisms. When these defence mechanisms are inadequate, the oxidative stress can damage proteins, carbohydrates, lipids and nucleic acids. Natural antioxidants are safer than synthetic antioxidants. Many researchers have been searching for powerful but nontoxic antioxidants from natural sources, especially edible or medicinal plants [2]. Such natural antioxidants could prevent the formation of free radicals primarily reactive oxygen species (ROS) which are associated with the disorders.

Substances causing damage to DNA of a cell are known as genotoxins [3]. DNA damage is one of the most important consequence of oxidative stress in the cells. If DNA repair is unable to modify these inducible DNA damages, genomic instability may lead to mutation, cancer, aging and many other diseases [4]. In human blood lymphocytes the chromosomal aberrations, sister chromatid exchange and micronucleus formation are considered as biomarkers of exposure to carcinogenic agents and genotoxic changes. Some researchers hold that the underlying mechanisms of DNA damage are similar in different tissues, thus suggesting that damage levels in lymphocytes may reflect those occurring in other tissues [5]. Genotoxicity assays are designed to detect compounds that induce directly or indirectly damage the genetic material by different mechanisms, being a fundamental requirement for the assessment mutagenicity toxicological characterization of a chemical [6]. Comet assay or single cell gel electrophoresis (SSGE) assay is often used since it is fast, convenient and easy to apply among the variety of methods developed for detecting DNA damage. Because of its low cost and sensitivity researchers focused on this technique [7].

*Achillea millefolium* L. belongs to Asteraceae family and it is represented by about 85 species mostly found in Europe and Asia and a handful in North America. It is commonly known as Yarrow in English and has different vernacular name in Urdu (Brinjasuf). It possess anti-inflammatory, analgesic, antiulcer, anxiolytic, hepatoprotective, hypotensive, and antiproliferative against human tumoral cells [8].

*Chaerophyllum villosum* (family Apiaceae) is a herb and 60 cm tall. It is commonly known as Jangli gajar [9]. It is widely distributed in East Asia Himalayas comprising India, Nepal and China and it also propagates in humid and cold environments on the road sides or open areas at height ranging from 2100 to 3500 m. Plant can be useful to cure cough, cold, stomach pain. Since the genotoxic effect of *Achellia millefolium* and *Chaerophyllum villosum* is not assessed so far, thus the aim of the present study was to determine the genotoxic or genoprotective potential of methanolic and chloroformic extract of the subject plants on human blood lymphocytes via Comet assay [10].

## Materials and methods

### Plant collection

The fresh plants of *Achellia millefolium* and *Chaerophyllum villosum* were collected from Miranjani top (2,992 m), Nathia Gali, Khyber Pakhtunkhwa, Pakistan. These plants were authenticated by Mr. Ghulam Jelani, at the University Boys College, University of Peshawar, Pakistan. Voucher specimen numbers i.e., M. Adil Bot.2244 (PUP) and M. Adil Bot.2245 (PUP) was given and deposited in Herbarium, Department of Botany, University of Peshawar for future purpose.

### Extraction

The collected plant parts were cleaned and washed thoroughly with tap water. The garbled plant parts were then partially dried by fan aeration and then fully dried in the oven at below 40 °C for more than two weeks. The fully dried plant parts were then ground to a powdered form and stored in suitable condition for few days. The powdered plant material (500 g) was soaked in (1000 ml), (97% methanol and chloroform) for two weeks. Both extracts were passed through (Whatman filter paper No.1823). The resulting methanolic and chloroformic extracts were subjected to rotary evaporator at 40 ºC to get concentrated crude extracts.

### HPLC analysis

HPLC analysis of chloroformic and methanolic extract of *A. millefolium* and *C. villosum* was executed with the help of Shimadzu HPLC System (model LC-20AD). Binary solvent was used for delivery with Rheodyne type of injector consisting of 20µL loop of sample and SPD-M 20 DAD detector instrument. 1 mL/min was the value for flow rate while the sample and standard solution were kept at 20µL. Chromatography was done utilising reverse phase mechanism of separation using Capcell Pack c-18, 5 µm, 251 mm into 4.5 mm along with an guarded column of extended type. Non-stationary section consisted of acetonitrile-methanol-aqua in the ratio of 40;15;45 having acetic acid 1% with an elution in isocratic for about half an hour. Diode detector range was maintained between 240 and 800 nm. For data acquisition and processing technique Shimadzu LC software was used. Peaks were identified by comparing retention time and spectrograph obtained through UV analysis, with that of referenced standard.

### Quantitative phytochemical screening

The quantitative chemical tests of methanolic, chloroformic extracts of *Achillea millefolium* and *Chaerophyllum villosum* were performed in order to detect alkaloids, phenols, flavonoids and tannins by using standard procedures of [11].

### Genotoxic activity

The genotoxic activity was studied by following the protocol of [12] using comet assay to analyse the amount of breakage in DNA per cell. Greater the rate of movement of fragments of DNA greater will be the damage induced. Cells infused in agarose gel were arranged on a microscopic slide. It was then treated with a basic solution followed by sodium chloride in order to completely remove histone proteins. Strands of DNA were dyed with propidium iodide to better view their movement away from nucleus. Damaged and undamaged DNA were observed under a fluorescence microscope (Leica DMR) attached to CCD-300E camera scoring, at 40X magnification of objective lens, by image J software based on the length of the DNA head and tail. Human blood lymphocytes were donated by the principle author for comet assay in 100, 200 and 300 concentration. Using software Comet Assay IV (instrument Haverhill, UK) 100 cells per slide were analysed.

Total comet score was measured on the basis of tail length by using formula;

$$DI=\frac{\mathrm{Total}\;\mathrm{cells}\;C0+\mathrm{Total}\;\mathrm{cells}\;C1+2X\;\mathrm{Total}\;\mathrm{cells}\;C2+3X\;\mathrm{Total}\;\mathrm{cells}\;C3+4X\;\mathrm{Total}\;\mathrm{cells}\;C4}{\mathrm{Total}\;\mathrm{Number}\;\mathrm{of}\;\mathrm{Cells}\;\mathrm{Under}\;\mathrm{Obversation}}$$

where, DI = Damage Index

Undamaged cells were placed in Class 0

- C1 = class 1
- C2 = class 2
- C3 = class 3
- C4 = class 4

### Antioxidant activity

The antioxidant activity was studied by following the protocol of [13]. DPPH scavenging activity of chloroformic and methanolic extract of *A.millefolium* and *C.villosum* was carried out. Solution of 0.135 mM DPPH in methanol and chloroform (0.03–0.1 mg of plant extract) was prepared. This mixture of solution was left in dark conditions for about half an hour. Absorbance value of the solution was measured spectrophotometrically at 517 nm. Ascorbic acid was used as referenced standard in this assay. Scavenging activity percentage was calculated using the formula:

$$\%\;\mathrm S\mathrm c\mathrm a\mathrm v\mathrm e\mathrm n\mathrm g\mathrm i\mathrm n\mathrm g=\frac{\mathrm{Absorbance}\;\mathrm{control}-\mathrm{Absorbance}\;\mathrm{sample}}{\mathrm{Absorbance}\;\mathrm{control}}\times100$$

Where,

$$Absorbance\;control\:=\:absorbance\;of\;DPPH\:+\:methanol\;/\;chloroform$$

$$\mathrm{Absorbance}\;\mathrm{sample}=\mathrm{Absorbance}\;\mathrm{of}\;\mathrm{DPPH}+\mathrm{sample}\;\mathrm{extract}/\text{standard}$$

Calculation of IC_50_ values were determined by utilising RTCA software for Data Analysis (1.00 version).

### Statistical analysis

Data was analysed using SPSS version 20. One-Way ANOVA was used to compare the groups following Tukey’s test. Values were expressed as mean ± standard deviation (S.D). Difference significant relative to positive control at *P < 0.01, **P < 0.002, ***P < 0.001.

## Results & discussion

### Phytochemical screening

The quantitative analysis of *A. millefolium* showed that alkaloids were maximum (27.4%) in methanolic extract, tannins were maximum (14.6%) in chloroform and phenol (34.5%) and flavonoid (32.4%) were maximum in methanol extract (Table 11). Similarly, the quantitative chemical analysis of *Chaerophyllum villosum* revealed that alkaloids was maximum (31.4%) in chloroformic extract, tannins were maximum (26.4%) in methanolic extract while phenols (19.3%) and flavonoids (35.5%) were maximum in chloroformic extract (Table 11). Flavonoids helps in the inhibition of topoisomerase I and II enzyme thus enhancing the formation of cleaveable DNA enzyme complexes and inhibiting the relegation of DNA double strand breaks. Phenolic compounds are found responsible for DNA breaks and mutation [3].

### HPLC analysis

**Fig. 1:**
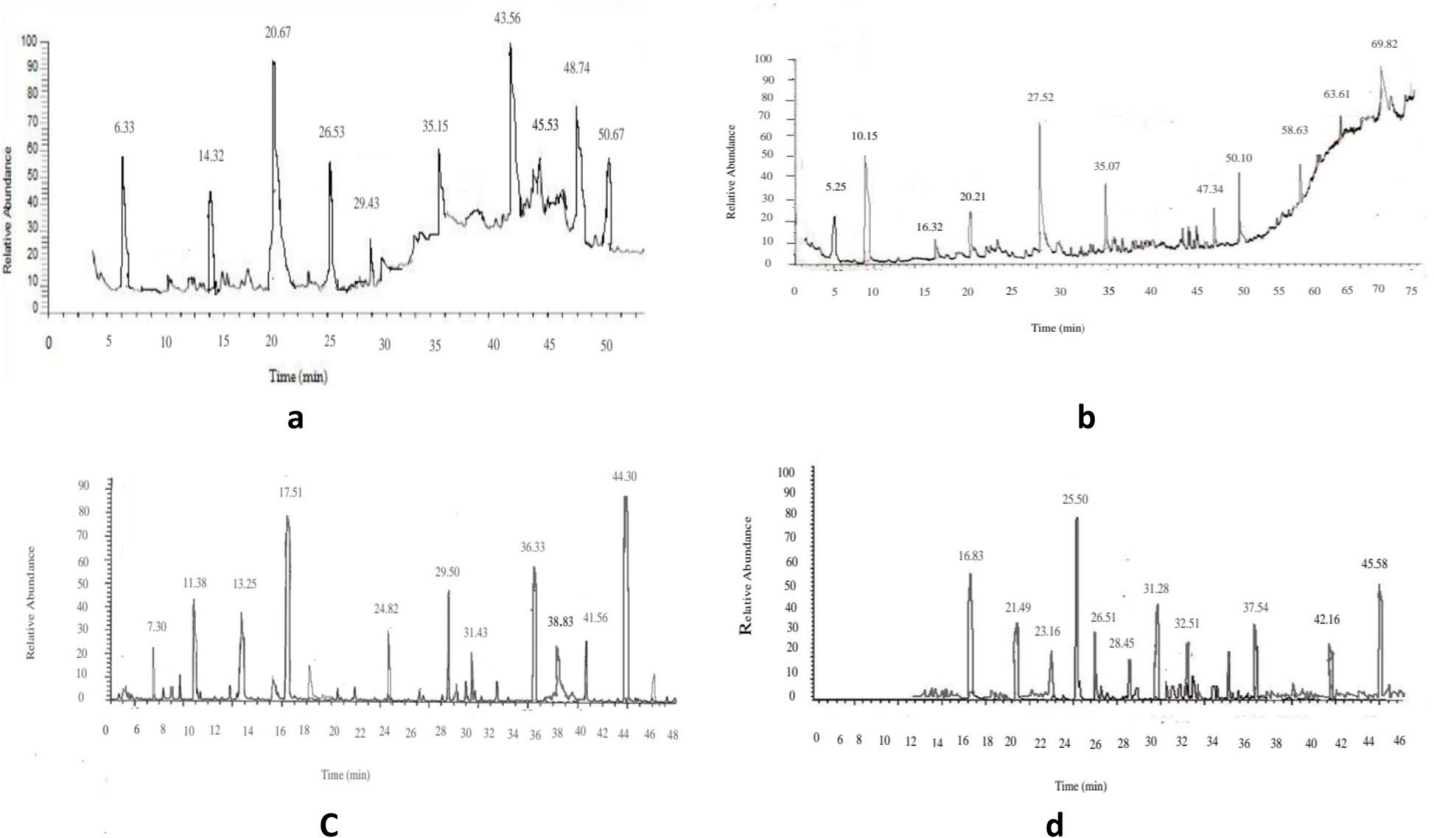
**A** HPLC chromatogram of methanol extract of *Achellia millefolium*. **B** HPLC chromatogram of chloroform extract of *Achellia millefolium*. **C** HPLC chromatogram of methanol extract of *Chaerophyllum villosum*. **D** HPLC chromatogram of chloroformic extract of *Chaerophyllum villosum*

HPLC analysis of methanolic and chloroformic extract of *A. millefolium* revealed the presence of six active constituents i.e. Apegenin, caffeic acid, kaempferol, syringic acid, ferulic acid, sinapic acid (Table 1A, Fig. 1A) and five active constituents i.e. quercitin, salicyclic acid, cinnamic acid, apeginin, rutin (Table 1B, Fig. 1B) respectively. While methanolic and chloroformic extract of *C.villosum* revealed the presence of kaempferol, myrcitin, caffeic acid, ellagic acid, catechin, rutin, chlorogenic acid (Table 1C, Fig. 1C) and luteolin, m coumaric acid, caffeic acid ellagic acid, ferulic acid, rutin (Table 1D, Fig. 1D) respectively.

**Table 1:** Quantitative phytochemical screening of *Achillea millefolium* L. and *Chaerophyllum villosum* Wall. ex DC

**Achillea millefolium L.**					
S.No	Extract	Alkaloids (mg/g)	Phenols (mg/g)	Tannins (mg/g)	Flavonoids (mg/g)
1	Methanol	27.4	34.5	12.7	32.4
2	Chloroform	16.6	18.6	14.6	25.6
**Chaerophyllum villosum Wall. ex DC.**					
1	Methanol	24.7	13.5	26.4	28.3
2	Chloroform	31.4	19.3	16.6	35.5
A: HPLC analysis of methanol extract of Achellia millefolium					
Peak no		Retention time	Compounds		Area
1		4.53	Apegenin		12453.21
2		10.32	Caffiec acid		21543.12
3		13.65	Kaempferol		42536.42
4		16.45	Syringic acid		52512.25
5		22.43	Ferulic acid		35231.13
6		26.36	Sinapic acid		64372.38
B: HPLC analysis of chloroform extract of Achellia millefolium					
Peak no		Retention time	Compounds		Area
1		11.42	Quercitrin		24262.41
2		18.63	Salicylic acid		43241.23
3		25.53	Cinnamic acid		65362.53
4		35.34	Apegenin		82142.71
5		43.51	Rutin		76722.43
C: HPLC analysis of methanol extract of Chaerophyllum villosum					
Peak no		Retention time	Compounds		Area
1		10.64	Kaempferol		25623.23
2		16.50	Myricetin		31352.21
3		24.43	Caffeic acid		54152.32
4		29.33	Ellagic acid		63143.38
5		36.42	Catechin		83125.62
6		39.31	Rutin		96543.41
7		44.52	Chlorogenic acid		92521.30
D: HPLC analysis of chloroform extract of Chaerophyllum villosum					
Peak no		Retention time	Compounds		Area
1		12.52	Luteolin		13673.43
2		23.41	m Coumaric acid		21435.32
3		30.62	Caffeic acid		42121.52
4		32.30	Ellagic acid		52174.21
5		40.24	Ferulic acid		64142.45
6		44.48	Rutin		72642.61

### Genotoxic activity

In the present research work the genotoxic and antigenotoxic potential of methanolic and chloroformic extract of *Achillea millefolium* and *Chaerophyllum villosum* were assessed on human lymphocytes DNA via comet assay. The genotoxicity test should be done to assess the potential for DNA damage such mutation, numerical changes or chromosomal recombination. Herbal extracts with positive genotoxicity test results may be indicative of potential carcinogenicity/mutagenicity risk to humans [14]. The results showed that maximum DNA impairment was detected in lymphocytes treated with standard hydrogen peroxide and methanolic extract of *Achillea millefolium* and *Chaerophyllum villosum* (78.0 ± 9.6), (47.7 ± 14.1), (38.3 ± 20.1) and (69.3 ± 5.8), (45.0 ± 1.7), (38.7 ± 4.0) at dosage of 50 mg/100 mL, 75 mg/100 ml and 100 mg/100 ml after 3 h (Tables 2 and 3). The genotoxicity of methanolic extract of *C. villosum* is higher than *A. millefolium* after 3 h. It indicated that methanolic extract of *C. villosum* after 3 h had maximum genotoxic agents. These results are in line with [15] who reported genotoxic effect of methanolic extract of *Pterolobium stellatum*. The methanolic extract of *Achellia millefolium* and *Chaerophyllum villosum* after 24 h exhibited dose dependent antigenotoxic activity (98.7 ± 12.7), (61.0 ± 3.6), (39.0 ± 3.6) and (85.7 ± 22.0), (54.7 ± 4.0), (44.0 ± 7.2) at 50 mg/100 ml, 75 mg/100 ml and 100 mg/100 ml and sequential reduction of total comet scoring and exhibited significant results (*p* > 0.0001) (Tables 4 and 5). Similarly, the maximum DNA damage calculated through tail length using Comet Assay IV softaware was observed in lymphocytes treated with standard hydrogen peroxide and chloroformic extract of *Achillea millefolium* and *Chaerophyllum villosum* (95.7 ± 19.0), (81.7 ± 16.6), (58.7 ± 35.8) and (71.7 ± 9.5), (42.0 ± 8.7), (31.7 ± 4.7) after 3 h (Tables 6 and 7). The chloroformic extract of *A. millefolium* and *C. villosum* after 24 h exhibited dose dependent antigenotoxic potential (116.0 ± 5.3), (66.7 ± 18.9), (59.6 ± 58.5) and (101.7 ± 8.6), (45.7 ± 9.0), (33.0 ± 4.3) at 50 mg/100 ml, 75 mg/100 ml and 100 mg/100 ml and successive decrease of total comet recording and displayed significant results (*p* > 0.0001) (Tables 8 and 9). It indicated that *Chaerophyllum villosum* methanolic extract is more potent in preventing DNA damage than *Achellia millefolium*. Similar results are reported by [16, 17] who reported antigenotoxic potential of *Olea europea* and *Gymnosporia Montana*.

**Table 2:** Comet assay of genomic DNA of human lymphocytes exposed to methanolic extract of *Achillea millefolium* L. for 3 h

	**Negative Control (Only Lymphocytes)**	**Positive Control (Lymphocytes + H**_**2**_**O**_**2**_**)**	**50 mg/100 ml**	**75mg100ml**	**100 mg/100 ml**
**Class 0**	90.0 ± 5.0	15.0 ± 5.0	54.3 ± 4.0	76.0 ± 5.3	81.0 ± 6.5
**Class 1**	4.0 ± 1.7	43.3 ± 11.5	23.3 ± 5.8	10.7 ± 1.2	8.7 ± 1.1
**Class 2**	3.3 ± 1.5	20.0 ± 13.2	12.3 ± 2.5	6.7 ± 2.8	4.7 ± 0.6
**Class 3**	1.7 ± 1.1	16.7 ± 7.6	6.0 ± 1.7	4.3 ± 1.5	2.7 ± 2.5
**Class 4**	1.0 ± 1.0	8.3 ± 2.8	4.0 ± 1.7	2.7 ± 1.1	3.0 ± 2.6
**TCS**	17.3 ± 13.1	166.7 ± 42.5	78.0 ± 9.6*	47.7 ± 14.1**	38.3 ± 20.1***

Values are expressed as mean ± standard deviation (S.D). Difference significant relative to positive control at *P < 0.01, **P < 0.002, ***P < 0.001 (One-way ANOVA, Tukey Test)

*TCS* Total comet score

**Table 3:** Comet assay of genomic DNA of human lymphocytes exposed to methanolic extract of *Chaerophyllum villosum* Wall. ex DC. for 3 h

Class	Negative Control (Only Lymphocytes)	Positive Control (Lymphocytes + H_2_O_2_)	50 mg/100 ml	75 mg/100 ml	100 mg/100 ml
**Class 0**	85.0 ± 5.0	12.3 ± 2.5	51.3 ± 1.5	69.0 ± 1.0	76.7 ± 4.0
**Class 1**	4.7 ± 0.6	25.0 ± 5.0	33.3 ± 1.5	21.7 ± 2.1	13.7 ± 4.6
**Class 2**	4.3 ± 1.2	31.7 ± 7.6	10.3 ± 2.1	6.0 ± 1.0	6.7 ± 2.1
**Class 3**	3.0 ± 2.0	20.0 ± 10.0	3.0 ± 1.0	2.0 ± 0.0	1.7 ± 0.6
**Class 4**	3.0 ± 2.0	11.0 ± 8.5	2.0 ± 1.0	1.3 ± 0.6	1.7 ± 0.6
**TCS**	34.3 ± 16.0	192.3 ± 16.6	69.3 ± 5.8*	45.0 ± 1.7*	38.7 ± 4.0*

Values are expressed as mean ± standard deviation (S.D). Difference significant relative to positive control at *P < 0.0001 (One-way ANOVA, Tukey Test)

*TCS* Total comet score

**Table 4:** Comet assay of genomic DNA of human lymphocytes exposed to methanolic extract of *Achillea millefolium* L. for 24 h

	**Negative Control (Only Lymphocytes)**	**Positive Control (Lymphocytes + H**_**2**_**O**_**2**_**)**	**50 mg/100 ml**	**75mg100ml**	**100 mg/100 ml**
**Class 0**	85.0 ± 5.0	12.3 ± 2.5	47.7 ± 2.5	63.7 ± 3.2	76.0 ± 5.2
**Class 1**	4.7 ± 0.6	25.0 ± 5.0	28.3 ± 2.8	20.0 ± 2.0	11.0 ± 3.6
**Class 2**	4.3 ± 1.2	31.7 ± 7.7	12.3 ± 2.5	10.3 ± 2.5	6.7 ± 2.1
**Class 3**	3.0 ± 2.0	20.0 ± 10.0	7.7 ± 2.5	3.7 ± 1.1	3.0 ± 1.0
**Class 4**	3.0 ± 2.0	11.0 ± 8.5	5.7 ± 1.1	2.3 ± 0.6	1.7 ± 0.6
**TCS**	32.7 ± 18.6	192.3 ± 16.6	98.7 ± 12.7*	61.0 ± 3.6*	39.0 ± 3.6*

Values are expressed as mean ± standard deviation (S.D). Difference significant relative to positive control at *P < 0.0001 (One-way ANOVA, Tukey Test)

*TCS* Total comet score

**Table 5:** Comet assay of genomic DNA of human lymphocytes exposed to methanolic extract of *Chaerophyllum villosum* Wall. ex DC. for 24 h

Class	Negative Control (Only Lymphocytes)	Positive Control (Lymphocytes + H_2_O_2_)	50 mg/100 ml	75 mg/100 ml	100 mg/100 ml
**Class 0**	90.0 ± 5.0	8.3 ± 5.7	50.6 ± 7.1	61.7 ± 13.7	73.7 ± 8.5
**Class 1**	6.0 ± 3.6	21.6 ± 10.4	27.3 ± 8.1	21.0 ± 9.6	16.3 ± 7.7
**Class 2**	3.0 ± 2.0	21.7 ± 7.6	14.3 ± 2.5	10.0 ± 2.0	6.3 ± 0.6
**Class 3**	0.3 ± 0.5	18.3 ± 7.6	6.3 ± 3.2	5.0 ± 1.7	2.3 ± 0.6
**Class 4**	0.6 ± 1.1	26.7 ± 12.6	2.7 ± 2.1	2.3 ± 0.6	1.3 ± 0.6
**TCS**	15.7 ± 6.6	226.7 ± 41.6	85.7 ± 22.0*	54.7 ± 4.0*	44.0 ± 7.2*

Values are expressed as mean ± standard deviation (S.D). Difference significant relative to positive control at *P < 0.001 (One-way ANOVA, Tukey Test)

*TCS* Total comet score

**Table 6:** Comet assay of genomic DNA of human lymphocytes exposed to chloroformic extract of *Achillea millefolium* L. for 3 h

	**Negative Control (Only Lymphocytes)**	**Positive Control (Lymphocytes + H**_**2**_**O**_**2**_**)**	**50 mg/100 ml**	**75mg100ml**	**100 mg/100 ml**
**Class 0**	90.0 ± 5.0	8.3 ± 5.7	48.3 ± 7.6	60.0 ± 13.2	68.3 ± 16.1
**Class 1**	6.0 ± 3.6	21.7 ± 10.4	25.0 ± 8.7	19.0 ± 9.6	13.3 ± 7.6
**Class 2**	3.0 ± 2.0	21.7 ± 7.6	13.3 ± 2.9	14.3 ± 6.0	10.0 ± 5.0
**Class 3**	0.3 ± 0.6	18.3 ± 7.6	9.3 ± 6.0	6.0 ± 3.6	5.7 ± 3.7
**Class 4**	0.7 ± 1.2	26.7 ± 12.6	4.0 ± 1.7	4.0 ± 1.7	2.7 ± 2.1
**TCS**	14.7 ± 8.4	226.7 ± 41.6	95.7 ± 19.0*	81.7 ± 16.6**	58.7 ± 35.8***

Values are expressed as mean ± standard deviation (S.D). Difference significant relative to positive control at *P < 0.003, **P < 0.002, ***P < 0.001 (One-way ANOVA, Tukey Test)

*TCS* Total comet score

**Table 7:** Comet assay of genomic DNA of human lymphocytes exposed to chloroformic extract *Chaerophyllum villosum* Wall. ex DC. for 3 h

Class	Negative Control (Only Lymphocytes)	Positive Control (Lymphocytes + H_2_O_2_)	50 mg/100 ml	75 mg/100 ml	100 mg/100 ml
**Class 0**	90.0 ± 5.0	15.0 ± 5.0	54.7 ± 2.5	75.0 ± 4.0	81.0 ± 2.6
**Class 1**	4.0 ± 1.7	43.3 ± 11.5	25.3 ± 4.9	13.3 ± 1.1	11.0 ± 1.7
**Class 2**	3.3 ± 1.5	20.0 ± 13.2	12.0 ± 2.6	7.7 ± 4.0	4.7 ± 0.6
**Class 3**	1.6 ± 1.1	16.7 ± 7.6	4.3 ± 1.1	2.7 ± 1.2	2.0 ± 0.0
**Class 4**	1.0 ± 1.0	8.3 ± 2.8	2.3 ± 1.5	1.3 ± 0.6	1.3 ± 0.6
**TCS**	19.7 ± 11.6	161.7 ± 48.5	71.7 ± 9.5*	42.0 ± 8.7**	31.7 ± 4.7***

Values are expressed as mean ± standard deviation (S.D). Difference significant relative to positive control at *P < 0.01, **P < 0.002, ***P < 0.001 (One-way ANOVA, Tukey Test)

*TCS* Total comet score

**Table 8:** Comet assay of genomic DNA of human lymphocytes exposed to chloroformic extract of *Achillea millefolium* L. for 24 h

	**Negative Control (Only Lymphocytes)**	**Positive Control (Lymphocytes + H**_**2**_**O**_**2**_**)**	**50 mg/100 ml**	**75mg100ml**	**100 mg/100 ml**
**Class 0**	92.7 ± 2.5	6.7 ± 2.8	42.7 ± 6.8	71.6 ± 7.6	84.0 ± 6.0
**Class 1**	3.3 ± 1.5	20.6 ± 9.0	21.7 ± 10.4	10.0 ± 5.0	10.0 ± 2.0
**Class 2**	1.3 ± 1.2	17.6 ± 7.5	15.0 ± 5.0	5.0 ± 5.0	3.0 ± 1.0
**Class 3**	2.3 ± 2.5	24.0 ± 5.3	11.7 ± 2.8	6.7 ± 2.8	1.3 ± 1.2
**Class 4**	0.3 ± 0.6	34.3 ± 4.0	7.3 ± 4.0	6.7 ± 2.8	1.3 ± 2.3
**TCS**	13.3 ± 8.5	265.3 ± 20.4	116.0 ± 5.3*	66.7 ± 18.9**	59.6 ± 58.5***

Values are expressed as mean ± standard deviation (S.D). Difference significant relative to positive control at *P < 0.002, **P < 0.0001, ***P < 0.0001 (One-way ANOVA, Tukey Test)

*TCS* Total comet score

**Table 9:** Comet assay of genomic DNA of human lymphocytes exposed to chloroformic extract of *Chaerophyllum villosum* Wall. ex DC. for 24 h

Class	Negative Control (Only Lymphocytes)	Positive Control (Lymphocytes + H_2_O_2_)	50 mg/100 ml	75 mg/100 ml	100 mg/100 ml
**Class 0**	92.7 ± 2.5	6.7 ± 2.8	45.7 ± 5.1	73.0 ± 5.0	78.7 ± 2.1
**Class 1**	3.3 ± 1.5	20.7 ± 9.0	24.3 ± 10.2	15.0 ± 3.0	13.0 ± 2.0
**Class 2**	1.3 ± 1.2	17.7 ± 7.5	17.3 ± 3.1	6.7 ± 1.5	5.7 ± 0.6
**Class 3**	2.3 ± 2.5	24.0 ± 5.3	8.0 ± 2.6	4.0 ± 1.7	2.0 ± 1.0
**Class 4**	0.3 ± 0.6	34.3 ± 4.0	4.7 ± 2.1	1.3 ± 1.1	0.6 ± 0.5
**TCS**	14.3 ± 8.6	265.3 ± 20.4	101.7 ± 8.6*	45.7 ± 9.0**	33.0 ± 4.3**

Values are expressed as mean ± standard deviation (S.D). Difference significant relative to positive control at *P < 0.002, **P < 0.0001 (One-way ANOVA, Tukey Test)

*TCS* Total comet score

### Antioxidant activity

The methanolic and chloroformic extracts of *A. millefolium* were studied for antioxidant activity by DPPH method. The DNA damage can be caused by genotoxicants either directly such as strand breaks, adducts, chromosome breakages, etc., or indirectly, by disturbing the genomic reliability through several mechanisms [18]. The H_2_O_2_ cause extensive oxidative damage when diffused to nucleus and form a hydroxyl radical and generate highly reactive oxygen and radical species by interacting with the transition metals bound to the DNA [19].The results revealed that methanolic extract of *Achillea millefolium* showed highest (84.21%) antioxidant activity as compared to the chloroformic extract (78.94%) at 300 mg/ml after 90 min but less than the standard drug ascorbic acid (88.72%). These results are comparable to that of methanolic and chloformic extract of *Momordica charantia* [20] and *Hygrophila auriculata* [21]. The lowest (IC_50_ = 58.98 µg/ml) was obtained for the methanolic extract (Table 10). The methanolic and chloroformic extracts of *C. villosum* were studied for antioxidant activity by DPPH method. The results showed that chloroformic extract of *Chaerophyllum villosum* exhibited highest (68.46%) antioxidant activity as compared to the methanolic extract (59.69%) at 300 µg/ml after 90 min but less than the standard drug ascorbic acid (88.72%). The lowest (IC_50_ = 57.16 µg/ml) was recorded for chloroformic extract (Table 11) using RTCA Data Analysis software. The antioxidant activity and antigenotoxic activity of plants might be due to the occurrence of phenols, flavonoids, alkaloids and tannins (Table 1). Hydroxyl groups of flavonoids participate in their antioxidant properties. The greater the number of OH groups on A and B rings of flavonoids, the higher is their antioxidant potential. Hydroxyl groups react strongly with free radicals making them unreactive [22]. Phenolic compounds add to the antioxidant potential of plants by neutralizing free radicals and avoiding decomposition of hydroperoxides into free radicals [23]. Tannins, specially, are characterized by a reductive chemical structure that has the capability for free radical appropriation [20]. Flavonoids have superoxide scavenging activities [24]. The antigenotoxic effect of these extracts against the mutagen agents may be attributed to the antioxidant effect of these extracts against the hydroxyl radicals, superoxide anions, and/ or their capacity to chelate or to stabilize transition metal ions, rendering them unable to participate in metal catalyzed initiation and radicals’ propagation. Iron-mediated formation of ROS leading to DNA and lipid damage appears to result from the amplification of the iron normal function, which is to transport oxygen to tissues [25]. This protective action of the tested extracts can be explained by their ability to penetrate the cell membrane and interrupt radical chain induced by H2O2, thus preventing and/or reducing free radical formation responsible for macromolecular damage, including DNA [26].

**Table 10:** Antioxidant activity of methanolic and chloroformic extract of *Achillea millefolium* L

Plant Extract	Conc. (µg/ml)	(%) DPPH radical scavenging activity					
		**30 min**	**60 min**	**90 min**	**Phenol contents (%)**	**Flavonoid contents (%)**	**IC**_**50**_** (µg/ml)**
Ascorbic acid	100	39.84	45.11	50.37	-	-	51.81
	200	57.14	63.9	71.42	-	-	
	300	62.40	78.94	88.72	-	-	
Methanol	100	30.0	36.84	45.86	34	32	58.98
	200	42.10	56.39	66.16			
	300	57.14	69.92	84.21			
Chloroform	100	36.09	42.10	46.61	18	25	63.60
	200	48.87	57.14	61.65			
	300	62.40	75.18	78.94

**Table 11:** Antioxidant activity of methanolic and chloroformic extracts of *Chaerophyllum villosum* Wall. ex Dc

Plant Extract	Conc. (µg/ml)	(%) DPPH radical scavenging activity					
		**30 min**	**60 min**	**90 min**	**Phenol contents (%)**	**Flavonoid contents (%)**	**IC**_**50**_** (µg/ml)**
Ascorbic acid	100	39.84	45.11	50.37	-	-	51.82
	200	57.14	63.9	71.42	-	-	
	300	62.40	78.94	88.72	-	-	
Methanol	100	27.0	37.59	43.60	13	28	79.28
	200	35.33	42.85	54.88			
	300	50.37	69.92	59.69			
Chloroform	100	34.58	43.60	47.36	19	35	57.16
	200	54.13	60.90	65.41			
	300	57.14	77.44	68.46			

## Conclusion

The results suggested that chloroform and methanol extracts of both plants showed anti-genotoxic potential, and antioxidant due to isolation of active compounds as apeginin, syringic acid, caffeic acid, kaempferol and ferulic acid. This study also confirmed that chloroform and methanol extracts of both plants were effective antioxidants which were achieved by the scavenging and chelating abilities observed against hydroxyl radicals or iron ions.

However further studies are needed to isolate the compounds of these plant species which will provide a better understanding of the genotoxic and antigenotoxic mechanisms described herein that might be helpful in research fields of aging process and age-related illnesses.

## Data Availability

The data such as the source file associated with this finding are available from the corresponding author upon request.
